# Focus on 2010 South African Heart Congress

**Published:** 2010

## Dangers of switching anti-hypertension medication

Physicians should discourage switching between anti-hypertension medications and should encourage both the patient and the funder to stick with the successfully up-titrated medication.

‘There is always the concern that by switching drugs, normally tolerated confidence intervals of bioequivalence studies may be compounded and result in poor blood pressure control and increased risk of suffering a cardiovascular or cerebrovascular event in at-risk patients.’ This view was presented in a very considered manner by Prof Peter Meredith, reader in clinical pharmacology at the University of Glasgow, who attended and spoke at the recent SA Heart Congress at Sun City

‘In my view, while most generics are quality products, the prescribing physician is entitled to get access to the relevant bioequivalence data – this is not done in the UK, Europe or the United States. At minimum, the comparator agent against which the generic has been shown to be bioequivalent should be indicated in the package insert.’

Evidence also suggests that it is quite difficult to mimic the nifedipine gastrointestinal therapeutic system (GITS),^1^,^2^ and the UK formulary still cautions against interchanging between nifedipine formulations. ‘The British National formulary states that different versions of modifiedrelease formulations may not have the same clinical effect. To avoid confusion between these different formulations of nifedipine, prescribers should specify the brand to be dispensed, Prof Meredith pointed out.

Dr Meredith stressed that no other modified-release preparation of nifedipine has demonstrated evidence of comparable clinical efficacy to nifedipine GITS in clinical outcome studies. ‘In my view also, none have demonstrated definitive evidence of bioequivalence to nifedipine GITS, as different bioequivalence standards apply in different regions. For example, in Europe, steady-state bioequivalence is required for modifiedrelease (MR) formulations, whereas this is not required for nifedipine MR formulations in the United States. However the FDA does require evidence of bioequivalence of nifedipine in both the fasting and fed situations, which is not a requirement in Europe.’

Research in Europe on different formulations^3^-^5^ has shown resultant differences in bioequivalence data, which have clinical implications for patient care, such as lack of 24-hour blood pressure control and the unnecessary addition of other medications to achieve targeted blood pressure levels. South African regulatory agencies require comparative bioavailability/ bioequivalence studies as proof of the efficacy of generic medicines but published guidelines are silent with regard to specific guidance concerning slow-release medications.

‘While the availability of generic medicines has played a major role in reducing drug costs and extending access, there are still far too many questionable generics worldwide’, Dr Meredith noted. ‘At a minimum, generic companies should show efficacy in the target population. Also, post-marketing surveillance of some kind should be done by non-innovator generic manufacturers as a contribution to self-inspection’, he concluded.
